# Interleukin-6: A Novel Target for Cardio-Cerebrovascular Diseases

**DOI:** 10.3389/fphar.2021.745061

**Published:** 2021-08-24

**Authors:** Jian-Hui Su, Meng-Yi Luo, Na- Liang, Shao-Xin Gong, Wei Chen, Wen-Qian Huang, Ying Tian, Ai-Ping Wang

**Affiliations:** ^1^ Institute of Clinical Research, Affiliated Nanhua Hospital, Hengyang Medical College, University of South China, Hengyang, China; ^2^ Hengyang Key Laboratory of Neurodegeneration and Cognitive Impairment, Department of Physiology, Institute of Neuroscience Research, Hengyang Medical College, University of South China, Hengyang, China; ^3^ Department of Anesthesiology, Affiliated Nanhua Hospital, Hengyang Medical College, University of South China, Hengyang, China; ^4^ Department of Pathology, First Affiliated Hospital, Hengyang Medical College, University of South China, Hengyang, China

**Keywords:** interleukin-6, inflammation, atherosclerosis, ischemic stroke, target, IL-6Rα

## Abstract

Cardio-Cerebrovascular Disease is a collective term for cardiovascular disease and cerebrovascular disease, being a serious threat to human health. A growing number of studies have proved that the content of inflammatory factors or mediators determines the stability of vascular plaque and the incidence of cardio-cerebrovascular event, and involves in the process of Cardio-Cerebrovascular Diseases. Interleukin-6 is a widely used cytokine that causes inflammation and oxidative stress, which would further result in cardiac and cerebral injury. The increased expression of interleukin-6 is closely related to atherosclerosis, myocardial infarction, heart failure and ischemic stroke. It is a key risk factor for these diseases by triggering inflammatory reaction and inducing other molecules release. Therefore, interleukin-6 may become a potential target for Cardio-Cerebrovascular Diseases in the future. This paper is aimed to discuss the expression changes and pathological mechanisms of interleukin-6 in Cardio-Cerebrovascular Diseases, and to provide a novel strategy for the prevention and treatment of Cardio-Cerebrovascular Diseases.

## Introduction

Cardio-Cerebrovascular Diseases (CCVDs) are the general term of all heart and brain diseases related to vascular diseases, such as atherosclerosis (AS), myocardial infarction (MI), heart failure (HF) and ischemic stroke (IS). CCVDs are common diseases that pose a serious threat to human health, especially to people over 50 years of age (Dalen et al., 2014). Despite efforts to reduce mortality, CCVDs remain the leading cause of death in the world (Reddy and Hunter, 2013). It is estimated that approximately 17.3 million people have died from CCVDs each year, accounting for nearly one third of global deaths; also, more than 80 percent of these deaths from CCVDs occur in developing countries (Piepoli et al., 2018; Polak et al., 2018). To conclude, CCVDs are characterized by high prevalence, high mortality and disability rates (Zhang et al., 2011), which not only put heavy economic pressure on families and society but also cause great physical or psychological suffering to individuals, directly adding to the global public health burden and hindering socio-economic development (Benjamin et al., 2019; Di Minno et al., 2019). In the face of such a critical situation, there is an urgent need to explore the pathogenesis of CCVDs as well as new therapeutic targets.

The pathogenesis of CCVDs is complex and multifactorial. CCVDs are known to be inflammatory diseases, and there is a strong link between inflammation and CCVDs (Soeki and Sata, 2016; Steven et al., 2019; Lu et al., 2021). Yudkin et al. Have shown that interleukin-6 (IL-6) and C-reactive protein (CRP) are key factors in the inflammatory reaction of CCVDs (Yudkin et al., 1999). IL-6 was first discovered and cloned in the 1980s by the Kishimoto’s laboratory (Hirano et al., 1989) as a small glycoprotein that can be produced by a variety of cells, and responds to various stimuli (Hirano, 1998). Members of the IL-6 family include IL-11, leukaemia inhibitory factor (LIF), oncostatin M (OSM), ciliary neurotrophic factor (CNTF), cardiotrophin-1 (CT-1), and novel neurotrophin-1/B cell stimulatory factor-3 (NNT-1/BSF-3) (Heinrich et al., 2003), sharing similar signaling pathways and playing important functions in inflammatory diseases, immune disorders and tumors. IL-6, an important member of this family, is considered to be an important pro-inflammatory factor (Kishimoto, 2005). IL-6 expression is tightly regulated, with low levels of expression in healthy individuals and elevated expression during infection, trauma or other stress. Zamani et al. demonstrated that IL-6 promoted the development and rupture of atherosclerotic plaques (Zamani et al., 2013). During myocardial ischemia-reperfusion, cardiomyocytes release IL-6, which induces neutrophils and cardiomyocytes to express CD11b/CD18 and intercellular adhesion molecule-1 (ICAM-1), thereby damaging the myocardium (Gwechenberger et al., 1999). In addition, IL-6 mRNA expression was elevated in a rat model of cerebral ischemia, and elevated IL-6 concentrations in the cerebrospinal fluid of patients with acute stroke was correlated with infarct volume (Tarkowski et al., 1995; Loddick et al., 1998). These studies suggest that the biological functions and mechanisms of IL-6 may play a key role in the pathogenesis of CCVDs.

Collectively, these data indicate that probing the biological functions and mechanisms of IL-6 may play a critical role in the pathogenesis of CCVDs. Our review provides a brief overview of the IL-6, reveals that IL-6 involves in the pathogenesis of CCVDs, and the therapeutic potential of IL-6 in CCVDs.

## Overview of Interleukin-6

### Secretion and Structure of Interleukin-6

IL-6 comes from a wide range of sources, mainly produced by mononuclear macrophages, T helper 2 cells, B cells, vascular endothelial cells (ECs), smooth muscle cells (SMCs) and fibroblasts (Schmidt-Arras and Rose-John, 2016). It acts not only on the immune system, but also on the neurological and cardiovascular systems. The human IL-6 gene is located on the short arm of chromosome 7, which is approximately 5 kb in length, including 5 exons and 4 introns. The molecule IL-6 is a glycoprotein with a molecular weight of 26 kDa, composed of 184 amino acids (Wolf et al., 2014), mediating a variety of biological effects by recognizing and binding to the IL-6 receptor (IL-6R) on the surface of target cells. IL-6R is widely expressed on the surface of various cells and consists of α and β polypeptide chains, namely, the specific binding α-subunit IL-6Rα and the signal-transducing β-subunit glycoprotein 130 (gp130) (Kishimoto et al., 1995). IL-6 has one binding site to IL-6Rα and two binding sites to gp130 (Paonessa et al., 1995) and the final formed ternary complex is a hexamer.

### Signal Pathway and Biological Function of Interleukin-6

**FIGURE 1:**
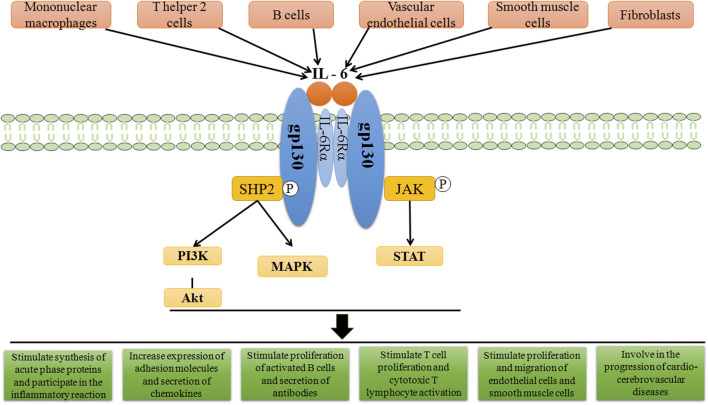
Schematic representation of IL-6 signal transduction. On target cells, IL-6 first binds to IL-6Rα, and subsequently associates with cellular membrane bound gp130, in turn inducing gp130 dimerization and initiation of IL-6 intracellular signalling. Formation of the IL-6/IL-6Rα/gp130 complex induces autophosphorylation and activation of the gp130-associated JAKs. These activated JAKs phosphorylate tyrosine residues within the cytoplasmic portion of gp130. Phosphorylation of the membrane-proximal tyrosine residue, leads to the recruitment of SHP2, which then stimulates the activation of MAPK and PI3K pathways. After the activation of JAKs, STAT molecules are recruited and then phosphorylated by the JAKs. The related down-stream proteins translocate afterwards into the nucleus to activate gene transcription, thereby playing the corresponding roles.

The combination of IL-6 and IL-6R can activate downstream signal transduction pathways (Figure 1). Through the engagement of unique receptor, IL-6 is able to activate Janus kinase (JAK)-STAT pathway, SHP2-mitogen-activated protein kinase (MAPK) pathway and phosphoinositide 3-kinase (PI3K)-protein kinase B (Akt) pathway (Garbers et al., 2015; Akbari and Hassan-Zadeh, 2018), among which, JAK1-STAT3 pathway is the main signaling pathway in the interleukin-6 family of cytokines (Taniguchi and Karin, 2014). IL-6 first binds to IL-6Rα to form a dimer, which is activated by phosphorylation, and then forms an activated trimer complex with gp130 (Rose-John, 2017), which initiates the intracellular signal cascade and induces the phosphorylation of JAK family related to IL-6R, further activating the downstream transcription factors of STAT family and binding to the promoter region of target genes, to generate a series of essential functions to maintain normal vital movements, such as inflammation, immune responses and cell recruitment (Table 1). Various IL-6 target genes are caused by activation of the transcription factor STAT3, which also stimulates the expression of genes encoding suppressor of cytokine signaling-1 (SOCS1) and SOCS3. In addition, SOCS1 can bind tyrosine-phosphorylated JAK, whereas SOCS3 binds tyrosine-phosphorylated gp130 to terminate IL-6 signaling through a negative feedback loop (Naka et al., 1997). The biological functions mediated by IL-6 are very complicated, which mainly include: 1) After the organism is stimulated by tissue injury, infection and inflammation, IL-6 could induce the production of cytokines including acute phase reaction proteins *via* NF-κB (Gauldie et al., 1987). 2) By activating endothelial cells and increasing the expression of adhesion molecules and the secretion of chemokines, IL-6 induces neutrophils to regroup into the affected tissue (Romano et al., 1997). 3) After antigen stimulating B cells, IL-6 could induce B cells to proliferate, differentiate and produce antibodies (Yao et al., 2014). 4) IL-6 could also induce the proliferation and differentiation of thymic T cells, activate macrophages (Chen et al., 2017) and natural killer cells, and participate in the coordination of immune system to resist harmful stimuli. 5) IL-6 is related to tissue fibrosis and vascular endothelial injury, promotes angiogenesis and increases vascular permeability (Tanaka et al., 2018) by stimulating the proliferation and migration of circulating endothelial progenitor cells (Fan et al., 2008), and also participates in the proliferation and migration of SMCs (Ikeda et al., 1991). Once IL-6 levels are abnormally elevated, the physiological disorder would occur, which leads to a series of pathological changes including inflammatory injury, plaque formation and rupture, and thrombosis. These changes has a promotional effect on the development of CCVDs.

**TABLE 1:** Function of Interleukin-6.

Action object	Specific roles of Interleukin-6
Neuron	Accelerate β amyloid accumulation (Jordan et al., 2017); Increase cerebrovascular diseases
Bone Marrow	Promote early bone marrow stem cell production; increase osteoclast to promote bone resorption; increase angiogenesis; increase platelet (Jordan et al., 2017)
Cardiac	Increase vascular endothelial growth factor to increase angiogenesis; increase cardiovascular diseases (Jordan et al., 2017)
Liver	Induce synthesis of acute phase proteins such as C-reactive protein, serum amyloid A and hepcidin (Tanaka et al., 2018); decrease albumin and transferrin; increase Fibrinogen (Jordan et al., 2017)
Immune cells	Stimulate B cells to produce immunoglobulin (Schett et al., 2013) and antibody (Jordan et al., 2017); increase plasmablast; promote the expansion and activation of T cells (Kopf et al., 1994); decrease T regulatory cells differentiation (Mangan et al., 2006; Unver and McAllister, 2018)
Kidney	Increase kidney mesangial proliferation (Jordan et al., 2017)
Skin	Increase Keratinocytes; increase dermal fibroblast collagen (Jordan et al., 2017)

## Roles of Interlukin-6 in Cardio-Cerebrovascular Diseases

As an important mediator of the inflammatory reaction, IL-6 acts extensively in the cardio-cerebrovascular system and is involved in the development of cardio-cerebrovascular pathologies. The role of IL-6 and its specific mechanisms in CCVDs would be discussed (Table 2).

**TABLE 2:** Role of Interleukin-6 in cardio-cerebrovascular diseases.

Type of diseases	The source of IL-6	Expression of IL-6	The mechanism of IL-6 in cardio-cerebrovascular diseases
Atherosclerosis	Endothelial cells, smooth muscle cells, macrophages, and T cells (Barton, 1996)	Up-regulation	Activate JAK/STAT cascade to induce CRP production to stimulate leukocyte recruitment and promote inflammatory responses; Induce LDLR expression of macrophages to promote LDL uptake and foam cell formation (Schuett et al., 2009); Degrade the extracellular matrix, and make plaques prone to rupture (Suzuki et al., 2008)
Myocardial Infarction	Endothelial cells, smooth muscle cells, cardiomyocytes, macrophages, monocytes, and neutrophils (Tsutamoto et al., 1998; Fuchs et al., 2003)	Up-regulation	Through JAK/STAT cascade, mediate neutrophil infiltration and activation; Reduce Ca^2+^ concentration through NO; Stimulate vascular endothelium and induce cardiomyocytes to the express of ICAM-1; Increase the production of PAI in liver (Kinugawa et al., 1994; Shu et al., 2007)
Heart Failure	Endothelial cells, smooth muscle cells, cardiomyocytes, macrophages, monocytes, and neutrophils (Yamauchi-Takihara et al., 1995; Tsutamoto et al., 1998)	Up-regulation	By activating JAK/STAT pathway, aggravate mitochondrial dysfunction caused by oxidative stress; Induce ROS production by regulating mitophagy level; Reduce actin phosphorylation; Promote cardiac fibroblasts to synthesize collagen; Increase nitric oxide synthase to decrease nitric oxide-mediated calcium flux and contractility in ventricular myocytes (Kinugawa et al., 1994; Huo et al., 2021)
Ischemic Stroke	Neurons, oligodendrocytes, astrocytes, and endothelial cells (Martinez-Revelles et al., 2008; Lambertsen et al., 2012)	Up-regulation	Affect phospholipid metabolism and produce arachidonic acid-like substances, ceramides and ROS (Adibhatla et al., 2008); Activate glial cells and cause leukocyte activation to infiltrate the CNS; Induce inflammatory immune cascade response via JAK/STAT pathway (Huang et al., 2006)

### Interlukin-6 and Atherosclerosis

AS is an inflammatory disease (Lusis, 2000) characterized by progressive lipid accumulation in the arterial wall, inflammation, SMCs infiltration and extracellular matrix remodeling that can lead to multiple clinically CCVDs (Ross, 1993), such as coronary artery disease, stroke and peripheral arterial diseases (Herrington et al., 2016). AS begins with endothelial damage, and inflammation is involved in the entire process of AS (Moriya, 2019). In 1994, Seino et al. found that IL-6 was expressed locally in coronary atherosclerotic plaques and in the walls of atherosclerosis-damaged arteries, and the expression was 10–40 times higher than in normal tissue (Seino et al., 1994). It has been demonstrated that IL-6 promoted the development and rupture of atherosclerotic plaques (Zamani et al., 2013), accelerating the progress of AS.

**FIGURE 2:**
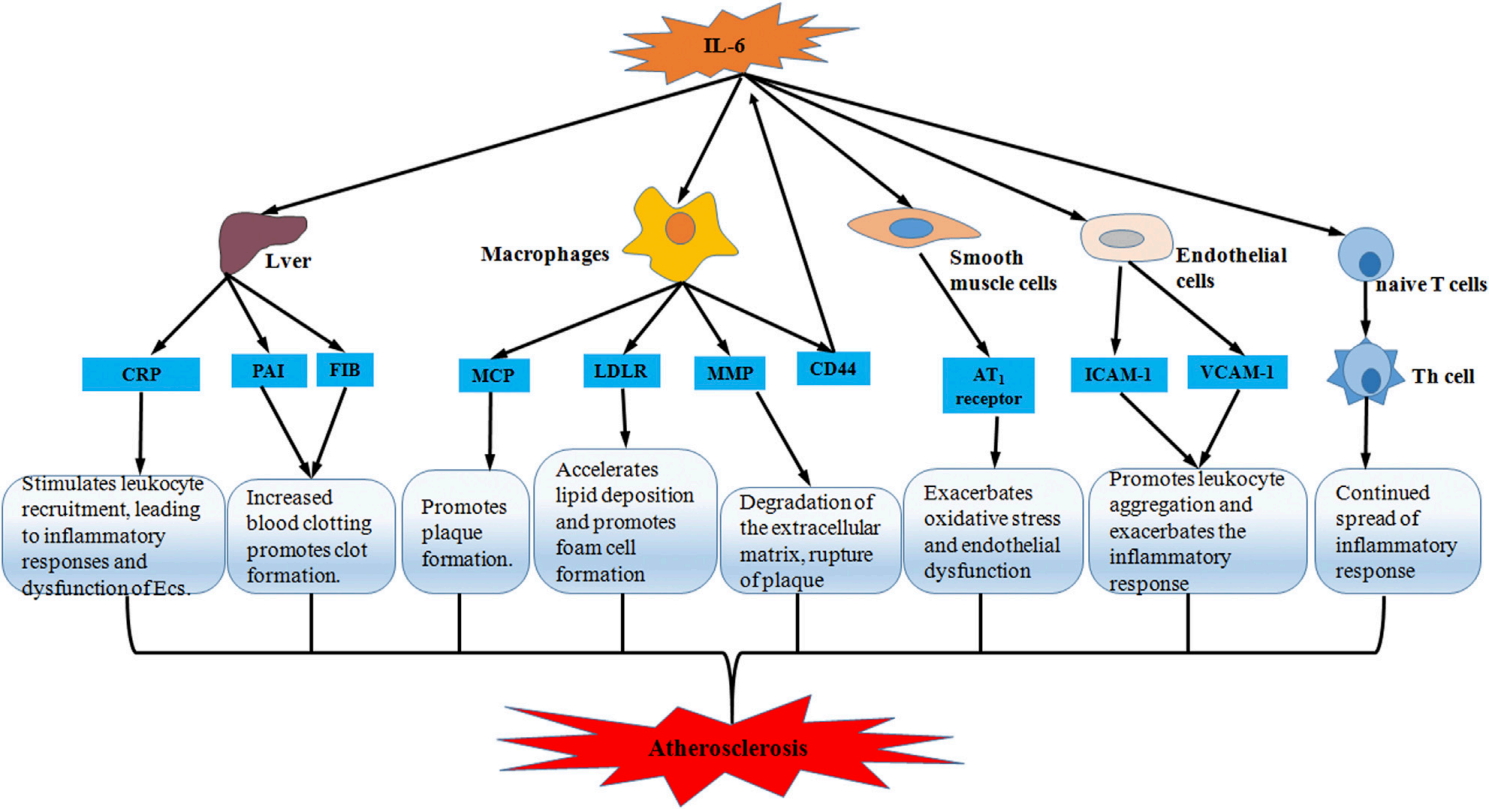
Schematic representation of IL-6 accelerating atherosclerosis. Through five pathways including liver, macrophages, smooth muscle cells, endothelial cells and T lymphocytes, IL-6 promotes inflammatory response and oxidative stress, and accelerates lipid deposition and foam cell formation, thereby participating in thrombosis and plaque formation and predisposing plaques to rupture. Ultimately, IL-6 exacerbates the progression of atherosclerosis.

IL-6 has a great many functions, including stimulating hepatic synthesis of acute phase reactants, activating endothelial cells, increasing coagulation, and promoting lymphocyte proliferation and differentiation (Wang et al., 2019). The different effects act on the different stages of AS would influence the development, progression and complications of AS (Figure 2). There are several possible pathways that are thought to be involved in the formation and progression of AS by IL-6: 1) IL-6 induces CRP production in the liver to stimulate leukocyte recruitment and promote inflammatory responses in endothelial cells, leading to endothelial dysfunction (Souza et al., 2008). 2) IL-6 induces plasma fibrinogen activator inhibitor (PAI) and fibrinogen production in hepatocytes, which increases blood clotting and promotes thrombosis (Koh et al., 2005; Darvall et al., 2007). 3) IL-6-activated macrophages secrete monocyte chemotactic proteins (MCP) to recruit monocytes into the subendothelium to participate in plaque formation (Lee et al., 2005). 4) IL-6 induces low-density lipoprotein receptor (LDLR) expression on the surface of macrophages to promote macrophage uptake of low-density lipoprotein (LDL), accelerating lipid deposition and promoting foam cell formation (Schuett et al., 2009). 5) IL-6 increases the expression of the cell adhesion molecule CD44 in macrophages, however, the high expression of CD44 also increases the secretion of IL-6 from macrophages in a positive feedback loop that exacerbates the progression of AS (Hägg et al., 2007). 6) IL-6 increases matrix metalloproteinase (MMP) synthesis, degrades the extracellular matrix, and makes plaques prone to rupture (Madan et al., 2008; Suzuki et al., 2008). 7) IL-6 increases the expression of vascular cell adhesion molecule-1 (VCAM-1) and ICAM-1, and promotes leukocyte aggregation to exacerbate the inflammatory reaction (Hartman and Frishman, 2014). 8) IL-6 increases angiotensin II type 1 (AT1) receptor expression in vascular SMCs which exacerbates oxidative stress and endothelial insufficiency that promotes AS progression (Wassmann et al., 2004). 9) IL-6 promotes the differentiation of naive T lymphocytes into helper T lymphocytes (Th) that sustain the spread of the inflammatory reaction (Eddahri et al., 2009). The above findings suggest that IL-6 plays a vital role in the pathology of AS and contributes to the development of AS through multiple pathways.

### Interlukin-6 and Myocardial Infarction

MI refers to the ischemic heart disease of cardiomyocyte injury and death caused by acute and persistent ischemia and hypoxia of coronary artery. After MI, there would exist irreversible necrosis due to hypoxia in cardiomyocytes, followed by infiltration of inflammatory cells including neutrophils and monocytes, releasing pro-inflammatory factors such as IL-6 (Gwechenberger et al., 1999), activating their own immune systems, and resulting in severe inflammatory reaction.

One of the causes of MI is the accumulation of atherosclerotic plaques in the coronary artery (Iuchi et al., 2018). Vascular inflammation plays a crucial role in the formation and progression of plaque and rupture of fibrous cap which would trigger the local thrombosis and hypoxia-related myocardial injury (Ridker, 2016). IL-6 signaling is related to the initiation and destabilization of plaque (Schieffer et al., 2004), and also linked to the adverse consequences of acute ischemia (Lindmark et al., 2001). Thus, we speculate that there is a relationship between IL-6 and MI pathogenesis. Recent studies (Huang et al., 2015) have shown that myocardial remodeling after MI has become the main cause of infarct mortality. IL-6 mediates myocardial remodeling, cardiomyocyte apoptosis and reduction of myocardial contractility by inducing the collection of inflammatory cells in injured myocardium (Lee et al., 2005; Souza et al., 2008), which is proved once again that IL-6 plays a promoting role in the process of MI. It is reported that IL-6 increased in patients with acute myocardial infarction and reached a peak in about 3 days (Miyao et al., 1993; Marx et al., 1997; Ridker and Lüscher, 2014). In addition, in the animal model, it was found that the serum IL-6 in mice increased significantly within 6 h after myocardial ischemia/reperfusion (Iuchi et al., 2018). This evidence suggests that there is a positive correlation between IL-6 and the risk of myocardial infarction (Held et al., 2017). The level of IL-6 may be an important factor affecting the degree of MI and subsequent cardiac remodeling and function (Wang et al., 2018).

According to increasingly related reports and studies, the specific role of IL-6 in MI appears to be gradually clear. Cardiomyocytes produce IL-6 under hypoxic and ischemic stress, express in the infarct border zone after MI (Fuchs et al., 2003), activate JAK/STAT cascade through abundant gp130 signal transduction receptor in cardiomyocytes (Miyao et al., 1993) to exert negative inotropic and cytotoxicity (Finkel et al., 1992), and through the inflammatory reaction to mediate neutrophil infiltration and activation (Tsutamoto et al., 1998), release more kinds of cytokines into the blood, co-stimulating vascular endothelium and inducing cardiomyocytes to express ICAM-1 (Shu et al., 2007) to lead to myocardial fibrosis and ischemia/reperfusion injury (Gwechenberger et al., 1999), which accelerate myocardial damage and dysfunction (Halawa et al., 1999). In a word, the inflammatory reaction of MI is related to the induction of cytokines such as IL-6, and the synthesis of IL-6 is an indispensable part of ischemia/reperfusion injury response (Kukielka et al., 1995).

### Interlukin-6 and Heart Failure

HF is a syndrome of cardiac circulatory disorder caused by blood stasis in venous system and insufficient blood perfusion in arterial system due to the cardiac systolic and/or diastolic dysfunction. It is not an independent disease, but the final stage of heart disease progression. Immune activation and inflammation contribute to the initiation and development of HF (Gullestad et al., 2012). Inflammation is one of the key pathophysiological mechanisms of HF. In HF, a variety of cells produce inflammatory mediators like IL-6, which is not only a sign of inflammatory activation, but also may induce systolic dysfunction, ventricular dilatation, cardiomyocyte hypertrophy and apoptosis through different mechanisms, directly acting on the pathological process of HF (Yndestad et al., 2006), promoting the progress and deterioration of HF.

During HF, there are hemodynamic changes and oxidative stress, which is a powerful inducer of inflammatory cytokine IL-6 (Ozova et al., 2007). According to Matsumori (Matsumori et al., 1994), IL-6 levels increased in patients with HF. At the same time, some studies have shown that IL-6 spillover in peripheral blood circulation increased with the severity of HF (Tsutamoto et al., 1998; Ruiz-Ruiz et al., 2006). Additionally, the activity of IL-6 was also markedly increased in HF, accompanied by an increase in gp130 level (Fischer and Hilfiker-Kleiner, 2007). In the HF model constructed by ligating the left anterior descending branch of coronary artery in rats, ischemia and hypoxia promoted the production of IL-6 (Kang et al., 2008). The evidence fully showed that the expression of IL-6 in blood circulation and myocardium from patients with HF was increased, and the level of IL-6 in blood circulation was bound up with the HF progress. Ventricular remodeling mediated by inflammation is an important cause for the onset and deterioration of HF (Mann, 2002), in which IL-6 regulates the whole inflammatory process and promotes the development of ventricular remodeling (Huang et al., 2015). The increase of cardiac IL-6 and IL-6R mRNA levels is also associated with hemodynamic deterioration in patients with advanced HF (Plenz et al., 2001). Moreover, there is a significant correlation between IL-6 and HF-related mortality (Maeda et al., 2000). Hence, as one of inflammatory mediators involved in HF, IL-6 triggers and aggravates HF by mediating myocardial remodeling, reducing myocardial contractility and promoting cardiomyocyte apoptosis. The increase of IL-6 expression is in connection with the decrease of coronary flow reserve, ejection fraction and cardiac function, and the progression of HF.

As the activation of inflammatory pathway is a crucial pathological event in the occurrence and development of HF (Mann, 2002), and IL-6 is one of the most well-characterized and principal cytokines in cardiovascular disease (Askevold et al., 2014), understanding the specific role of IL-6 in HF counts for the target therapy in HF. Under the stimulation of ischemia and hypoxia, IL-6 binds to its receptor-coupled protein gp130 by autocrine or paracrine, and then transduces signals into cells *via* JAK/STAT3 signaling pathway, which induces cardiomyocyte hypertrophy and leads to abnormal endothelium-dependent vasodilation (Tsutamoto et al., 1998), muscular atrophy (Tsujinaka et al., 1996) and left ventricular dysfunction (Fuchs et al., 2003). Finkel et al. (Finkel et al., 1992) found that IL-6 induced nitric oxide-mediated decrease in calcium flux and contractility in ventricular myocytes (Kinugawa et al., 1994) by increasing nitric oxide synthase (Gwechenberger et al., 1999), which eventually caused ventricular remodeling. Meléndez et al. (Meléndez et al., 2010) found that IL-6 promoted cardiac fibroblasts to synthesize collagen and led to cardiac interstitial fibrosis, which in turn caused ventricular wall sclerosis and HF. IL-6 can also increase the stiffness of cardiomyocytes by reducing actin phosphorylation (Markousis-Mavrogenis et al., 2019). To sum up, increased IL-6 could be regarded as a considerable independent predictor for HF (Orús et al., 2000), and could also be utilized as a prognostic biomarker in HF (Rauchhaus et al., 2000).

### Interlukin-6 and Ischemic Stroke

IS is one of the leading causes of death and disability worldwide (Wang, 2005). Inflammation plays a key role in the progression of IS (Barone and Feuerstein, 1999; Chamorro and Hallenbeck, 2006), yet the underlying mechanisms are largely unknown. Studies have shown that cerebral ischemia can disrupt the dynamic balance between pro- and anti-inflammatory responses, and suppressing the inflammatory reaction can reduce brain damage and improve neurological function (Yilmaz and Granger, 2008). IL-6 is low expressed in normal brain tissue but significantly elevated in response to injury, infection, stroke, and inflammation (Herrmann et al., 2003). IL-6 is produced by neurons, oligodendrocytes, astrocytes, and vascular ECs during cerebral ischemia (Suzuki et al., 1999; Martinez-Revelles et al., 2008; Lambertsen et al., 2012). Microglia are rapidly activated during cerebral ischemia and become key cells in the inflammatory reaction in the brain, secreting pro-inflammatory cytokines such as IL-6, IL-1β and TNF-α, which are involved in microglia-mediated neurological injury (Lambertsen et al., 2012). Previous studies have indicated that IL-6 was elevated during IS (Herrmann et al., 2003). IL-6 has several potentially essential functions in the pathogenesis of stroke. Some studies have found that cytokines such as TNF-α, IL-1α/β and IL-6 affect phospholipid metabolism during acute inflammatory reaction under cerebral ischemia, producing arachidonic acid-like substances, ceramides and reactive oxygen species (ROS), which can cause damage to the brain tissue (Adibhatla et al., 2008). After vascular occlusion, IL-1 and IL-6 expression increases and acts on vascular endothelial cells to express ICAM-1, P-selectin, and E-selectin, causing leukocyte aggregation and adhesion and mediating the inflammatory cascade to worsen cerebral ischemic injury (Huang et al., 2006).

In summary, IL-6 is one of the inflammatory cytokines in central nervous system (CNS) that activates glial cells and causes leukocyte activation to infiltrate CNS (Carlson et al., 1999). IL-6 causes inflammatory damage during cerebral ischemia and its pathological mechanisms including induction of chemotactic factor expression and synthesis of intracellular adhesion molecules would lead to an inflammatory immune cascade response, in conjunction with a compromised blood-brain barrier that causes leukocyte infiltration.

### Interlukin-6 and Other Cardio-Cerebrovascular Diseases

**FIGURE 3:**
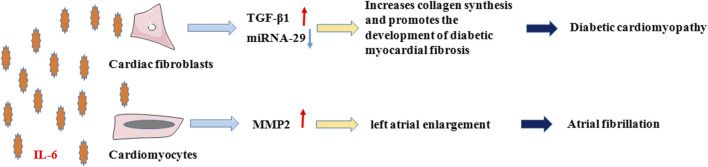
IL-6 in Diabetic cardiomyopathy and Atrial fibrillation. In diabetic cardiomyopathy and atrial fibrillation, on the one hand, IL-6 promotes the development of diabetic myocardial fibrosis by enhancing TGF-β1 and inhibiting the expression of miRNA-29 in cardiac fibroblasts, which increases collagen synthesis. On the other hand, IL-6 promotes the secretion of MMP-2 from cardiomyocytes, which causes atrial fibrillation due to enlargement and remodeling of the left atrium.

In addition to the four most predominant diseases in the clinic, IL-6 can also affect diabetic cardiomyopathy (DCM) and atrial fibrillation (AF) (Figure 3). DCM is one of the major cardiovascular complications of diabetes by impairing the diastolic and systolic functions of the heart, which eventually leads to HF (Borghetti et al., 2018). The pathogenesis of DCM involves varied mechanisms, of which myocardial fibrosis has been shown to be the main pathological change that reduces cardiac compliance and stiffens heart by producing excess collagen (Westermeier et al., 2016). Meléndez et al. (2010)) found that IL-6 promoted collagen synthesis in cardiac fibroblasts and led to cardiac interstitial fibrosis. Zhang et al. suggested that IL-6 promoted the development of diabetic myocardial fibrosis by enhancing TGF-β1 and inhibiting miRNA-29 expression, thereby increasing collagen synthesis, and that (Zhang et al., 2014) knockdown of the IL-6 gene attenuated the development of myocardial fibrosis in DCM.

AF is a common clinical arrhythmia with a complex pathogenesis that is not yet fully understood. A meta-analysis (Wu et al., 2013) showed that high plasma IL-6 levels were associated with a high risk of AF in the general population and of AF recurrence following electrical cardioversion and radiofrequency ablation therapy. Aulin et al. (2015) concluded that in patients with AF, high IL-6 level is related to thromboembolism and major bleeding and is an independent risk factor for thromboembolic events. Although the mechanism of IL-6 in the pathogenesis of AF is not clear, studies (Marcus et al., 2008) have shown that IL-6 promotes MMP-2 secretion and is linked to left atrial enlargement, which is a known risk factor for AF. Therefore, it is hypothesized that IL-6 may cause AF through left atrial remodeling.

## The Therapeutic Potential Role of IL-6 in Cardio-Cerebrovascular Diseases

IL-6 has been shown to play a vital role in chronic inflammatory diseases (Tanaka and Kishimoto, 2012) which is closely related to CCVDs. Down-regulation of IL-6 signaling is considered as a strategy to reduce cardiovascular risk. Secondary analysis from CANTOS suggests that the therapeutic benefit of IL-1 inhibition for cardiovascular disease is associated with a reduction in IL-6 levels (Ridker et al., 2020). Also, it has been proved that the level of IL-6 was increased in patients with stroke and was correlated with stroke severity (Lambertsen et al., 2012). Thus, suppressing the expression of IL-6 may be a potential target for the treatment of CCVDs.

### In Atherosclerosis

IL-6 signaling is in connection with atheromatous plaque formation and instability (Yudkin et al., 2000), and plasma IL-6 levels are used as a marker for cardiovascular diseases such as coronary artery disease and AS (Kinlay and Egido, 2006). In animal experiments, IL-6 administration to ApoE^-/-^ mice fed with normal or high-fat diet was found to exacerbate AS (Huber et al., 1999). In addition to the high expression of IL-6, an inducer of STAT3 in atherosclerotic plaques, activation of STAT3 was also detected in plaques (Recinos et al., 2007), and was associated with the progression of atherosclerotic lesions (Grote et al., 2005). STAT3 activation and atherosclerotic lesion development can be inhibited by using an anti-mouse IL-6 receptor antibody (MR16-1) (Akita et al., 2017). Pro-inflammatory cytokines are known for affecting both the expression of scavenger receptors and the formation of foam cells (Li and Glass, 2002). IL-6 is expressed in atherosclerotic lesions in macrophage-rich areas (Schieffer et al., 2000) and stimulates the inflammatory reaction of macrophages, as well as SMC proliferation and thrombogenic activity (Rattazzi et al., 2003). The administration of IL-6 inhibitor (Am80) in ApoE^-/-^ mice was able to suppress scavenger receptor expression and foam cell formation *in vitro* and prevent AS formation *in vivo* (Takeda et al., 2006). A few studies have also shown that IL-6 played a key role in angiotensin II (Ang II)-mediated CD36 expression and Ox-LDL uptake (Huber et al., 1999; Keidar et al., 2001). Consistently, CD36 obtained from ventral macrophages of IL-6-deficient mice is not up-regulated by Ang II stimulation (Keidar et al., 2001). In brief, these findings suggest that IL-6 is a major factor in plaque formation and instability, and that IL-6 promotes the development of AS, suggesting that inhibition of IL-6 or its receptors may be a novel approach to the prevention and treatment for AS.

### In Myocardial Infarction

IL-6 promotes platelet aggregation and thrombosis by stimulating the production of PAI in liver (Sakamoto et al., 1992), up-regulates the expression of ICAM-1 in cardiomyocytes to increase the release of oxygen free radicals, and accelerates the decrease of Ca^2+^ concentration (Kinugawa et al., 1994) through NO, thereby damaging cardiomyocytes and playing a negative role in MI. In contrast, Jing et al. (2019) demonstrated that inhibition of IL-6 gene expression could activate M2 macrophages, induce macrophage polarization and inhibit fibroblast activation to reduce collagen deposition, notably improving myocardial remodeling caused by MI. Held et al. (Held et al., 2017) also found that subcutaneous injection of IL-6 antibody could ameliorate left ventricular dysfunction.

### In Heart Failure

In HF patients, the increased IL-6 level is a predictive indicator of cardiac function deterioration and is in linkage to the poor prognosis of HF patients (Hirota et al., 2004). Up-regulated IL-6 promotes cardiac hypertrophy by aggravating mitochondrial dysfunction caused by oxidative stress through gp130/STAT3 signaling pathway, and induces excessive ROS production by regulating mitophagy level and increasing mitophagy-related protein expression, consequently exaggerating cardiomyocyte apoptosis (Huo et al., 2021). Besides, IL-6 changes Ca^2+^-handling and reduces myocardial contractility (Villegas et al., 2000), resulting in diastolic disturbance and arrhythmia (El-Adawi et al., 2003; Askevold et al., 2014). The accumulation of IL-6 provides an available inflammatory microenvironment for cardiac remodeling and subsequent HF. For this, suppressing the expression of IL-6 in myocardium may be a new way to treat HF. An experiment (Smart et al., 2006) has proved that the gene deletion of IL-6 is linked to the decrease of cardiac hypertrophy and fibrosis after angiotensin II stimulation in mice model. Plus, Kobara et al. (2010) found that the administration of MR16-1 could alleviate left ventricular remodeling after coronary artery ligation in LDLR^-/-^ mice. Similarly, in the TAC-induced mice model, IL-6 deletion attenuated left ventricular hypertrophy and dysfunction (Zhao et al., 2016). Meanwhile, as an inhibitor of IL-6/gp130, raloxifene could ameliorate myocardial remodeling in TAC mice, attenuate HF, and partially maintain cardiac function at the late stage (Huo et al., 2021).

Currently, direct evidence concerning IL-6 inhibition on the alleviation of Cerebrovascular Diseases is scarce, but based on the findings described in the previous section, IL-6 is significantly elevated during IS and is involved in the development of IS. Since IL-6 is a very important mediator that is one of inflammatory cytokines in CNS and leads to inflammatory damage, thus, it is reasonable to infer that IL-6 has significant implications for the prevention and treatment of Cerebrovascular Diseases such as IS.

As for the role of IL-6 inhibitors in CCVDs, there are some correlative studies have proved their effects. For instance, Tocilizumab (Actemra), the first humanized monoclonal antibody against IL-6R, plays protective roles by inhibiting the binding of IL-6 to IL-6R and blocking signal transduction, thereby reducing inflammation. Besides, Cheng et al. (Cheng et al., 2015) found that during ischemia/reperfusion injury, tocilizumab competitively bound to IL-6R, suppressed STAT phosphorylation, inhibited cardiomyocyte apoptosis and maintained cardiomyocyte activity by up-regulating the apoptosis-related factor Bax and down-regulating the expression of BCL-2. Additionally, Kleveland et al. (Kleveland et al., 2016) showed that tocilizumab inhibited the myocardial inflammatory reaction in patients with non-ST-elevation MI, reduced hs-CRP levels and decreased troponin T release. Moreover, Broch et al. (Broch et al., 2021) demonstrated that the early administration of IL-6 receptor inhibitor such as tocilizumab on patients with non-ST-elevation MI within 6 h of symptom onset could reduce myocardial necrosis and infarct size. Also, tocilizumab has been well tolerated and has no significant safety concerns. Hence, there is reason to believe that inhibitors of IL-6 have a protective effect against Cardiovascular Diseases.

## Conclusion and Prospect

**FIGURE 4:**
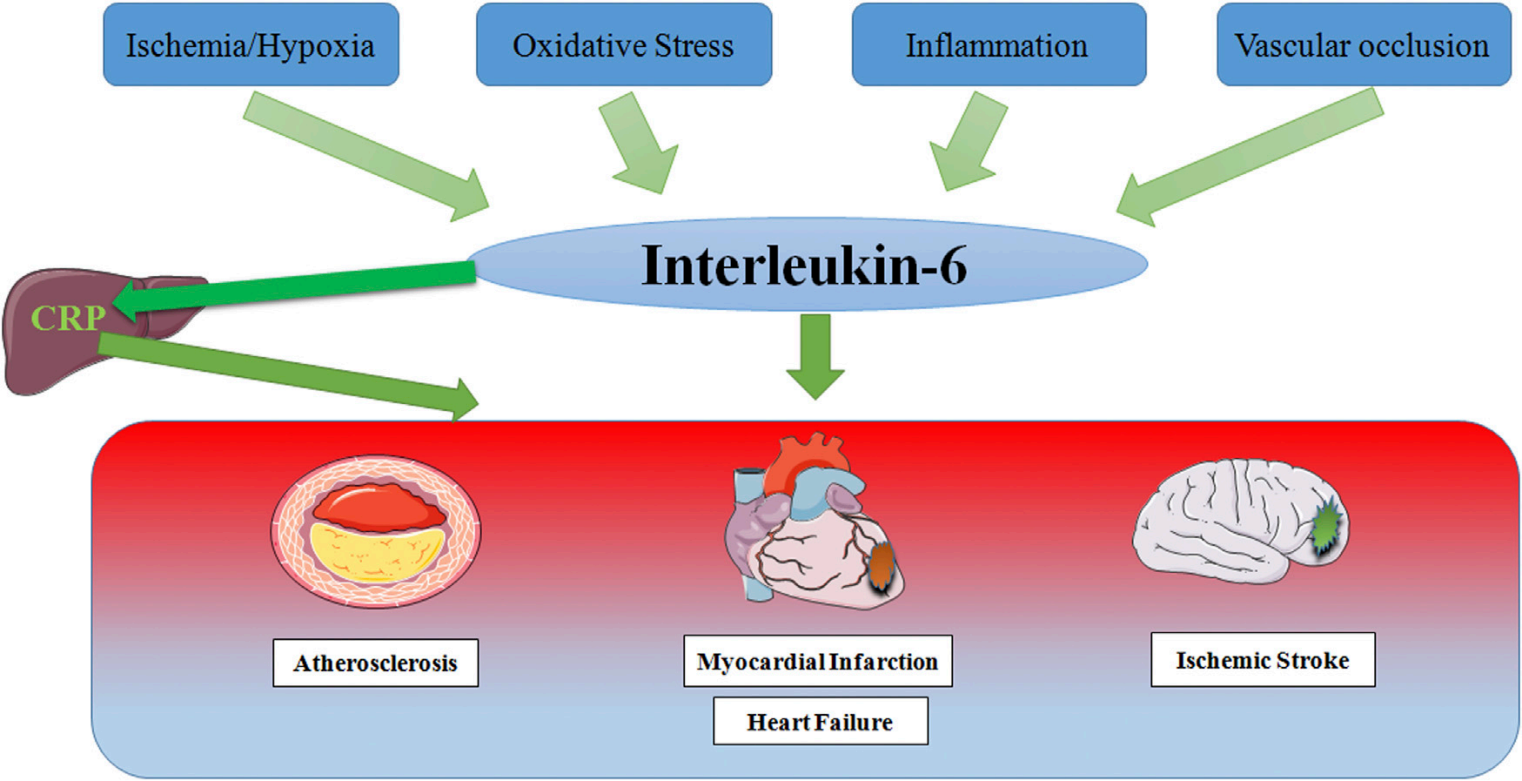
Schematic representation of risk factor inducing IL-6 in Cardio-Cerebrovascular Disease. In response to stimulation by ischemia and hypoxia, oxidative stress, inflammation and vascular occlusion, IL-6 levels are elevated, which partly leads to the production of the acute phase protein CRP in the liver, thereby stimulating leukocyte recruitment and thrombosis, ultimately causing multiple cardio-cerebrovascular diseases.

Since the discovery of IL-6, there have been numerous studies on the relationship between IL-6 and various diseases, and major research results have been achieved. Growing studies have made it clear that the level of IL-6 increases in varying degrees in CCVDs including AS, MI, HF and IS (Figure 4), and participates in the occurrence and development of CCVDs under the stimulation of ischemia, hypoxia, oxidative stress, inflammation and vascular occlusion. Inflammation underlies various physiological and pathological processes (Medzhitov, 2008), and its role in CCVDs is one of the hotspots of recent research. As one of the most considerable defense mechanisms *in vivo*, inflammation helps our body to fight against infection, injury and tissue destruction. However, once the inflammation is out of control or exists for a long time, a large number of inflammatory cells would gather in the vascular lesion sites, release potential toxic substances, directly damage the heart and brain tissue, finally resulting in cardiomyocyte and neuron edema or even death. Furthermore, inflammation is often accompanied by oxidative stress that generates excessive ROS. The strong oxidative activity is also able to damage normal cell structure, and promotes the progress of CCVDs. Moreover, inflammation during acute phase may directly affect thrombosis (Della Corte et al., 2016). Macrophages, a kind of immune cells, can penetrate damaged vascular endothelium to promote plaque formation and vascular wall fibrosis in the case of immune disorder and excess inflammation, then resulting in vascular lumen occlusion and aggravating ischemia/reperfusion injury. Cytokines release during inflammation also has the ability to alter the normal anticoagulant and pro-fibrinolytic properties of endothelium, especially IL-6, which accelerates fibrinogen production through signal transduction, accordingly promoting thrombosis and reducing myocardial contractility (Janssen et al., 2005).

IL-6 is an upstream cytokine that reflects the inflammatory reaction (Moriya, 2019), and is also a candidate biomarker for predicting CCVDs risk. By binding to and interacting with its specific receptor IL-6R, IL-6 activates signal transduction receptor complex gp130, and plays a cascade effect on Cardio-Cerebrovascular system, thence participates in CCVDs. In Cardiovascular Diseases, some researchers (Zhang et al., 2005) found that the level of IL-6 in myocardial tissue from MI rats increased greatly. After drugs were given to suppress the IL-6 expression, myocardial collagen deposition decreased and cardiac function was improved. Withal, a study (Harris et al., 1999) has manifested that there is a correlation between elevated IL-6 levels and cardiovascular mortality. Likewise, in Cerebrovascular Diseases, IL-6 may play a vital role in the diagnosis and prognosis of IS and could be used to predict the size of brain damage (Ferrarese et al., 1999; Lambertsen et al., 2012). The increase of IL-6 is closely associated with the size of cerebral infarction and the degree of neurological impairment. In the acute stage of cerebral ischemia, IL-6 would further aggravate ischemic injury. In conclusion, IL-6 involves in CCVDs by regulating inflammatory reaction. Although part of the mechanism is not clear yet, it can be predicted that the role of IL-6 in CCVDs would provide a new theoretical basis for the diagnosis, treatment and prognosis evaluation of diseases.

Wherefore, based on the above research foundation, in the future Cardio-Cerebrovascular therapy, IL-6 blockade strategy may provide a new therapeutic potential (Tanaka et al., 2016). Delving into the study concerning relationship between IL-6 and CCVDs and respective pathogenesis, is of great importance for the prevention and treatment of CCVDs. It is believed that with the deepening of related studies and the continuous improvement of detection technologies, anti-IL-6-related drugs or inhibitors would have a broad therapeutic prospect.
